# ECG trained artificial intelligence for the detection of patients with inducible myocardial ischemia

**DOI:** 10.1093/ehjdh/ztag050

**Published:** 2026-03-20

**Authors:** Jaehyun Lim, Gibeom Park, Hak Seung Lee, Joon-Myoung Kwon, Heesun Lee, Bongwon Suh, Hyun-Jae Kang, Yong-Jin Kim, Bon-Kwon Koo, Hyo-Soo Kim

**Affiliations:** Department of Internal Medicine, Seoul National University Hospital, 101 Daehak-ro, Jongno-gu, Seoul 03080, Republic of Korea; Department of Internal Medicine, Seoul National University College of Medicine, Seoul, Republic of Korea; Department of Intelligence and Information, Seoul National University, Seoul, Republic of Korea; Digital Healthcare Institute, Sejong Hospital, Bucheon, Republic of Korea; Medical Research Team, Medical AI, Seoul, Republic of Korea; Digital Healthcare Institute, Sejong Hospital, Bucheon, Republic of Korea; Medical Research Team, Medical AI, Seoul, Republic of Korea; Healthcare System Gangnam Center, Seoul National University Hospital, Seoul, Republic of Korea; Department of Intelligence and Information, Seoul National University, Seoul, Republic of Korea; Department of Internal Medicine, Seoul National University Hospital, 101 Daehak-ro, Jongno-gu, Seoul 03080, Republic of Korea; Department of Internal Medicine, Seoul National University College of Medicine, Seoul, Republic of Korea; Department of Internal Medicine, Seoul National University Hospital, 101 Daehak-ro, Jongno-gu, Seoul 03080, Republic of Korea; Department of Internal Medicine, Seoul National University College of Medicine, Seoul, Republic of Korea; Department of Internal Medicine, Seoul National University Hospital, 101 Daehak-ro, Jongno-gu, Seoul 03080, Republic of Korea; Department of Internal Medicine, Seoul National University College of Medicine, Seoul, Republic of Korea; Department of Internal Medicine, Seoul National University Hospital, 101 Daehak-ro, Jongno-gu, Seoul 03080, Republic of Korea; Department of Internal Medicine, Seoul National University College of Medicine, Seoul, Republic of Korea

**Keywords:** Inducible myocardial ischaemia, Artificial intelligence, Electrocardiogram, Screening

## Abstract

**Aims:**

Myocardial ischaemia is associated with adverse prognosis. Identifying high-risk individuals who require a stress test is challenging, and a practical screening tool to detect these patients, especially in asymptomatic individuals, is lacking. We aimed to develop an artificial intelligence (AI) model based on a resting 12-lead electrocardiogram to detect patients with inducible myocardial ischaemia.

**Methods and results:**

An AI model was developed using 12 074 resting 12-lead ECGs from 11 700 patients and tested on 1342 patients at two hospitals. Patients with inducible ischaemia were defined as those who received revascularisation for silent ischaemia, stable angina, or unstable angina between 2004 and 2020 (*n* = 6070). No ischaemia group included patients with 0% stenosis in all epicardial coronary arteries and coronary artery calcium score of ≤100 in coronary computed tomography angiography (*n* = 7346). The primary outcome was the model performance categorising patients with inducible myocardial ischaemia. We further validated the model through multiple reference and external validation datasets encompassing 35 898 patients. The model showed an area under the receiver operating characteristic curve (AUROC) of 0.90 (95% CI 0.88─0.92), and an area under the precision-recall curve (AUPRC) of 0.87 (95% CI 0.84─0.89). The model performance was robust regardless of age, sex, comorbidities, clinical diagnosis, or culprit vessels. Consistent results were demonstrated in an age- and sex-matched dataset (*n* = 7414; AUROC 0.85, 95% CI 0.83─0.87 and AUPRC 0.84, 95% CI 0.82─0.87), as well as in reference and external cohorts.

**Conclusion:**

Electrocardiogram-trained AI demonstrated favourable performance in detecting inducible myocardial ischaemia. It may enable screening and risk stratification of high-risk patients.

## Graphical Abstract

**Graphical Abstract:**
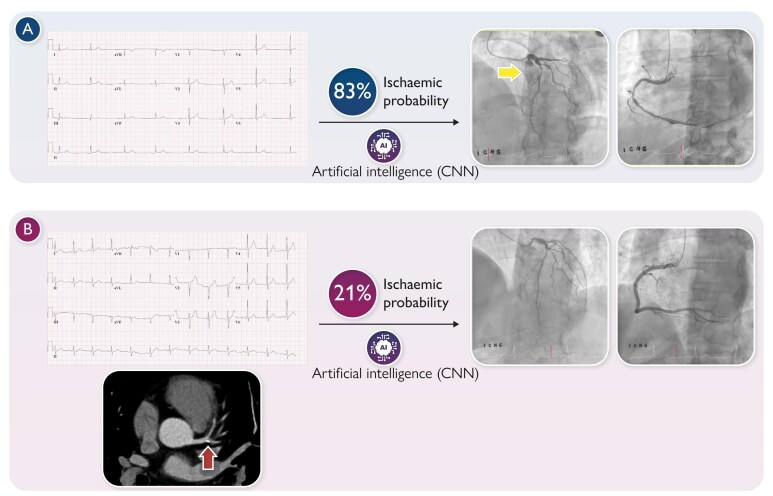

## Introduction

Ischaemic heart disease (IHD) is a chronic and most often progressive disease-causing substantial disability and mortality worldwide.^1^ Myocardial ischaemia is a phenomenon occurring in the myocardium as a result of inadequate supply of myocardial oxygen for the demand and is commonly associated with angina or myocardial infarction. However, it is not uncommonly encountered in the absence of symptoms. Identifying patients with inducible ischaemia is crucial due to its association with adverse prognosis regardless of the presence of symptoms.^2–4^ Although several approaches have been explored, screening strategies for high-risk patients who require further evaluation for inducible myocardial ischaemia remain challenging, particularly among asymptomatic individuals.^5–7^

Recent advancements in artificial intelligence (AI) are paving the way for novel applications in medicine.^8–13^ Notably, several studies that employed AI to interpret the electrocardiogram (ECG) have shown promise in identifying cardiac abnormalities that human cardiologists could not recognize through ECGs.^10–15^ AI analyses the entire ECG signal convolutionally, extracting complex, non-linear and interdependent features beyond traditional sequential human visual assessment.^9,10^ It may also detect fine details and faint signals at an analytical resolution surpassing human visual capabilities.

Previous literature has demonstrated several differences between patients with and without inducible ischaemia, even at rest, including various biomarkers or cell-level responses.^16,17^ In this study, we hypothesized that a properly trained AI model can identify differences in ECGs of patients with inducible myocardial ischaemia. To confirm this hypothesis, we developed and tested a supervised AI model based on resting 12-lead ECG.

## Methods

We adhered to the Standards for Reporting Diagnostic accuracy studies (STARD) 2015 guideline.^18^ The Seoul National University Hospital (SNUH) institutional review board approved the study.

### Definitions, data sources, and the study population

Optimal AI model training demands datasets that combine both substantial quantity and high label fidelity.^19^ We therefore defined two distinct patient groups: those with clearly evident inducible myocardial ischaemia and those without. For this purpose, we established comprehensive inclusion and exclusion criteria, aiming to encompass as broad a patient population as possible while preserving the clinical integrity of the ‘ischaemia’ and ‘no-ischaemia’ groups.

Although current guidelines suggest detecting inducible ischaemia with functional tests, discrepancies in results across modalities make any single test an unreliable ground truth for AI training.^20–22^ Therefore, the ‘ischaemia group’ included patients clinically diagnosed with silent ischaemia, stable angina, or unstable angina, and received coronary artery revascularisation on top of guideline-directed medical therapy. This pragmatic definition was chosen because the clinical decision to revascularize—supported by symptoms, positive functional tests, and high-grade angiographic stenosis—serves as a strong indicator of clinically significant myocardial ischaemia. Clinical diagnoses were derived from prospectively collected cohorts and/or individually reviewed. Patients with silent ischaemia were defined as asymptomatic patients with documented inducible ischaemia by functional tests or ≥90% stenosis on invasive coronary angiography (CAG). Functional tests included non-invasive exercise ECG, stress echocardiography, cardiac magnetic resonance imaging, myocardial single-photon emission computed tomography, and positron emission tomography, and invasive fractional flow reserve. Patients with symptoms and/or documented evidence of inducible ischaemia were classified as having stable angina. However, those presenting with accelerated angina (Braunwald classification I) were categorized as having unstable angina.^23^

**Figure 1:**
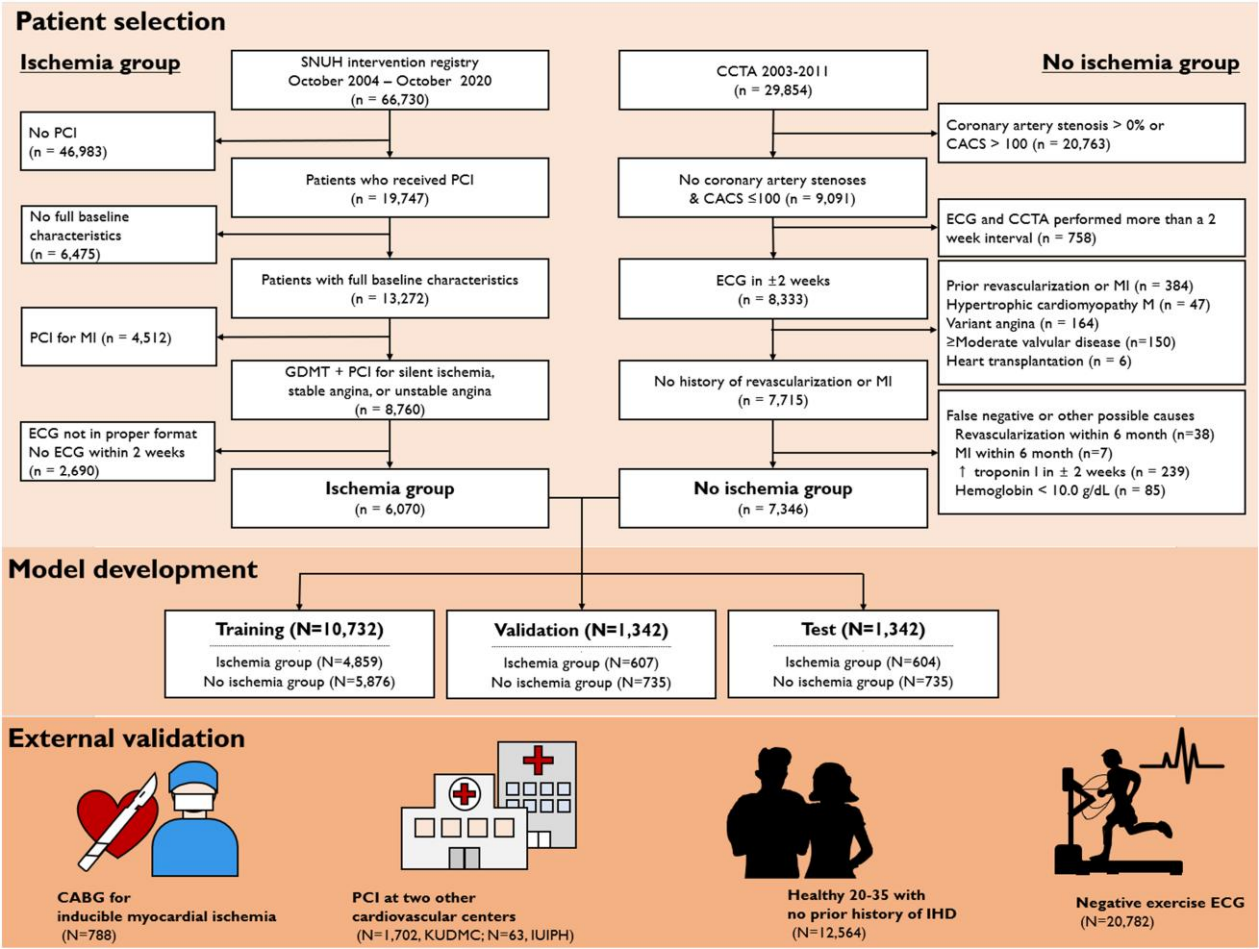
Study flow. CABG, Coronary artery bypass graft surgery; CACS, coronary artery calcium score; CCTA, coronary computed tomography angiography; ECG, electrocardiogram; GDMT, guideline-directed medical therapy; IUIPH, Inje University Ilsan Paik Hospital; KUDMC, Keimyung University Dongsan Medical Center; MI, myocardial infarction; PCI, Percutaneous coronary intervention; SNUH, Seoul National University Hospital.

The ‘no ischaemia group’ comprised patients with both 0% stenosis in all visible epicardial vessels and a coronary artery calcium score (CACS) of ≤100 on coronary computed tomography angiography (CCTA). This composite criterion was chosen to create a robust negative control, given the very high negative predictive values of 0% stenosis and CACS ≤100 for excluding meaningful epicardial coronary artery stenosis and inducible myocardial ischaemia, respectively.^24,25^ To preclude secondary or possible other causes of myocardial ischaemia, we excluded those with pre-existing other cardiac pathologies, a recent rise in troponin-I (>99th percentile) for any reason within 14 days, or a hemoglobin level below 10 g/dL. Additionally, those who underwent revascularisation or were diagnosed with MI within six months after CCTA were further excluded to preclude possible false-negative cases of the CCTA (*Figure 1*).

For inclusion in the ‘ischaemia group’, we initially identified 66,730 patients who underwent CAG at SNUH between October 2004 and October 2020. Of these, patients who received percutaneous coronary intervention (PCI) owing to silent ischaemia, stable angina, or unstable angina were identified. We selected the ECG recorded closest to the index PCI, obtained within two weeks prior to the procedure and, for symptomatic patients, after their symptom onset. Only one ECG closest to the date of PCI for each event was used. Vessels revascularized were considered the culprit vessels. The ‘no-ischaemia’ group was derived from two hospitals of SNUH and Healthcare System Gangnam Center, SNUH, between 2003 and 2011. One ECG per patient, performed within a 2-week interval and closest to the date of CCTA, was used.

The model was trained with digital, standard 10-second, 12-lead ECGs acquired in the supine position at a sampling rate of 500 Hz. All data were obtained in the XML format through the MUSE ECG system (GE Healthcare). Data from each group were randomly split into three parts: 80% for model training, 10% for internal validation, and another 10% for testing (*Figure 1*). Patients with multiple events were excluded from the test dataset to keep its independence. The model architecture is detailed in Supplementary material online, *Method S1* and Supplementary material online, *Figure S1*.

Analyses were performed using R software, version 4.2.2 (R Core Team), and the model was implemented with Python programming language, using the Keras framework with a Tensorflow (Google Inc.) backend.

### Study outcomes

The primary outcome was the performance of the model identifying patients with inducible myocardial ischaemia, assessed by calculating the area under the receiver-operating characteristic curve (AUROC), area under the precision-recall curve (AUPRC), and an F1-score in the test dataset. For each ECG input, the AI model produces an output value between 0 and 1; the optimal threshold was selected at the point that maximized the sum of sensitivity and specificity in the internal validation dataset. We used two-sided 95% confidence intervals (CIs) for sensitivities and specificities, using the Clopper-Pearson method. The 95% CIs for both the AUROC and AUPRC were generated using a bootstrapping method. Separate models trained exclusively with lead I, II, or a combination of these leads were also developed. Since the performance of the model could be affected by baseline differences in age and sex between the ischaemia vs. non-ischaemia groups, we also conducted a sensitivity analysis with a 1:1 age- and sex-matched dataset. The construction of this dataset is detailed in the Supplementary material online, *Method S2*.

The model performance was analysed by several subgroups categorized by age, sex, hypertension, or diabetes mellitus. Furthermore, test results of the ischaemia group were presented according to various subgroups, including clinical diagnosis, number of diseased vessels, culprit vessel, or type of functional test.

### Model validation on reference and external cohorts

We have validated the model using several independent, mutually exclusive cohorts.

Two different positive control groups with inducible myocardial ischaemia were used for validation. First, patients who underwent coronary artery bypass grafting (CABG) for silent ischaemia, stable angina, or unstable angina at SNUH between April 2005 and September 2020 were used as a reference standard for having inducible myocardial ischaemia. A single ECG per patient conducted within two weeks before and closest to the surgery was tested. Second, patients who received PCI for inducible ischaemia at two other cardiovascular centres, Keimyung University Dongsan Medical Center (KUDMC), and Inje University Ilsan Paik Hospital (IUIPH), were used for external validation: each hospital representing tertiary and secondary general hospitals, respectively.

For a negative control, ECGs of patients aged 19–35 without prior IHD diagnosis were used as a reference standard, given very low prevalence of IHD (<0.5%) in this age group.^26^ We also compared test results in individuals with negative exercise ECGs from routine health screenings at the Healthcare System Gangnam Center, SNUH.

## Results

### Study population

ECGs of 13 416 cases from 13 042 patients were used for the model development and validation: The ischaemia group consisted of 6070 cases from 5696 patients (mean age 66.2 ± 9.9 years, 71.2% men). Among these, approximately 85% (*n* = 5127) had ≥90% stenosis on CAG and/or positive functional test results, either performed invasively or non-invasively. The no-ischaemia group comprised 7346 patients (mean age 55.3 ± 9.8 years, 59.7% men). Baseline characteristics were similar across training, validation, and test datasets (see Supplementary material online, *Table S1*).

### Model performance

**Figure 2:**
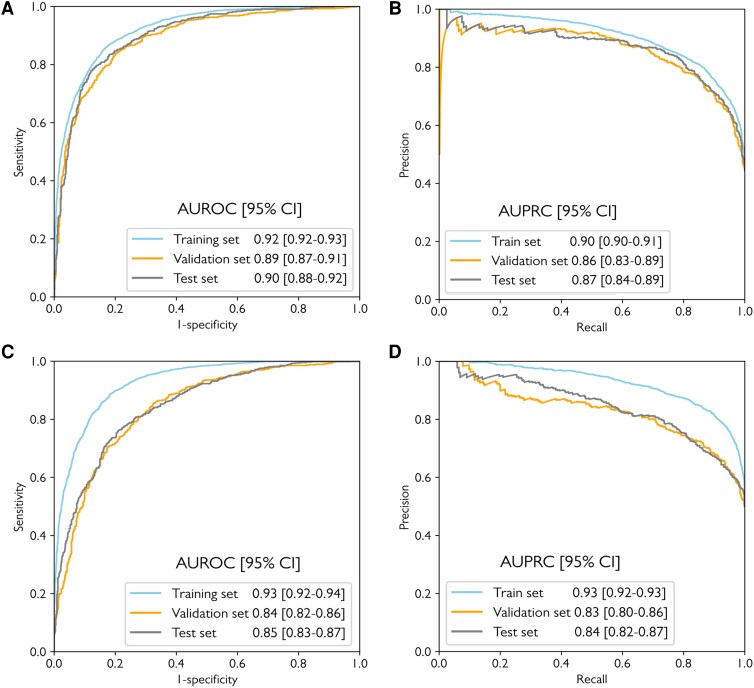
The model performance in the entire and 1:1 age- and sex-matched dataset. (*A*) AUROC and (*B*) AUPRC in the entire dataset. (*C*) AUROC and (*D*) AUPRC in the 1:1 age- and sex-matched dataset. AUPRC, area under the precision-recall curve; AUROC, area under the receiver operating characteristic curve; CI, confidence interval.

The model achieved an AUROC of 0.90 (95% CI 0.88─0.92), AUPRC of 0.87 (95% CI 0.84─0.89), and an F1-score of 0.82 in the test dataset (*Figure 2A* and *B*). At a threshold of 0.43, sensitivity was 83.8% (95% CI 80.6─86.6) and specificity was 79.6% (95% CI 76.5─82.5; *Table 1*, Supplementary material online, *Figure S2*). Single-lead models yielded an AUROC of 0.81 using lead I and 0.78 using lead II. When trained with both leads I and II, the AUROC improved to 0.84 (95% CI 0.82─0.86).

**Table 1:** Model performance on internal test, reference, and external validation datasets

Tested population	N	Test result
**Internal test dataset**		
Ischaemia group	604	506/604 (sensitivity 83.8%)
No ischaemia group	735	585/735 (specificity 79.6%)
**Reference and external validation datasets**		
**Positive controls**		
Patients who underwent CABG for inducible ischaemia	788	682/788 (sensitivity 86.5%)
Patients who received PCI for inducible ischaemia at KUDMC	1701	1376/1701 (sensitivity 80.9%)
Patients who received PCI for inducible ischaemia at IUIPH	63	54/63 (sensitivity 85.7%)
**Negative controls**		
Patients aged 19–35 with no prior history of ischaemic heart disease	12,564	11 590/12 564 (specificity 92.2%)
Patients with negative exercise ECG	20 782	17 543/20 782 (specificity 84.4%)

CABG, coronary artery bypass grafting; ECG, electrocardiogram; IUIPH, Inje University Ilsan Paik Hospital; KUDMC, Keimyung University Dongsan Medical Centre; PCI, percutaneous coronary intervention.

A model constructed using the age- and sex-matched dataset (*n* = 7414; baseline characteristics in Supplementary material online, *Table S2*) demonstrated an AUROC of 0.85 (95% CI 0.83─0.87) and AUPRC of 0.84 (95% CI 0.82─0.87; *Figure 2C* and *D*).

### Subgroup analysis

**Figure 3:**
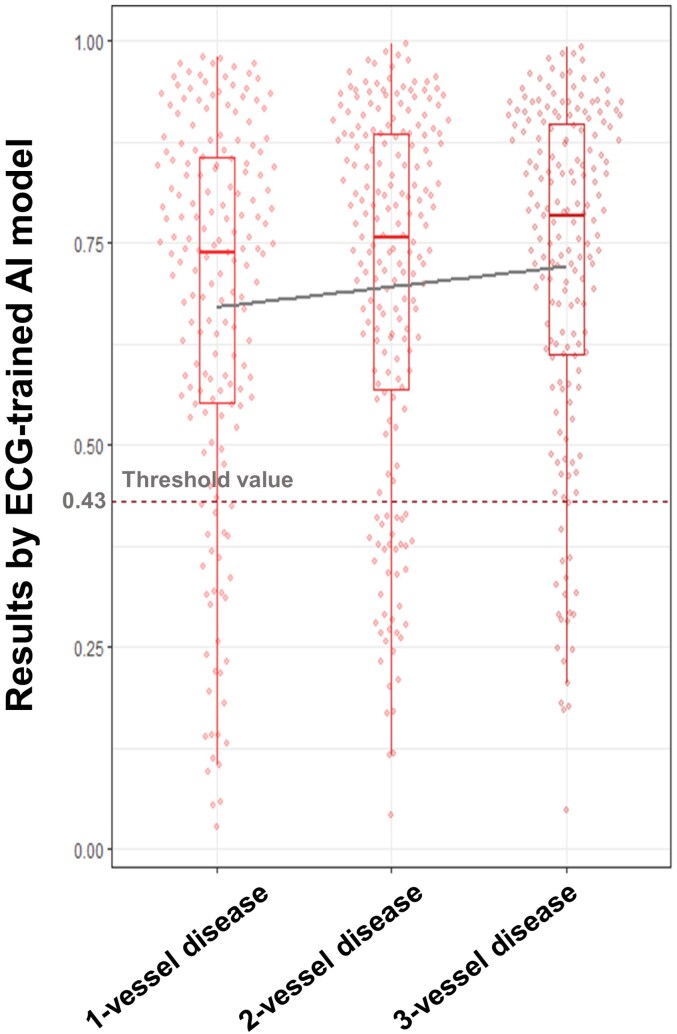
The distribution of results by ECG-trained AI according to the number of diseased vessels. A trend toward a higher sensitivity was shown in patients with a higher number of diseased vessels (*P*-value for trend = 0.053). AI, artificial intelligence; ECG, electrocardiogram.

Model performance was consistent across subgroups by age, sex, hypertension, or diabetes (see Supplementary material online, *Table S3*) and did not differ by clinical diagnosis or angiographic characteristics (*Table 2*). However, a trend towards higher sensitivities was observed with more diseased vessels (*P*-value for trend = 0.053, *Figure 3*). Subgroup analysis of the model generated from the matched dataset is presented in Supplementary material online, *Table S4*.

**Table 2:** Model performance by patient characteristics among the ischaemia group of the test dataset

	Test positive	Number of patients	Sensitivity (95% CI)
**Clinical diagnosis**			
Silent ischaemia	62	79	78.5 (67.8─86.9)
Stable angina	297	345	86.1 (82.0─89.6)
Unstable angina	147	180	81.7 (75.2─87.0)
**n-vessel disease**			
1-vessel disease	146	178	82.0 (75.6─87.4)
2-vessel disease	160	199	80.4 (74.2─85.7)
3-vessel disease	162	185	87.6 (81.9─92.0)
Left main disease	38	44	86.4 (72.6─94.8)
**Culprit vessel in a 1-vessel disease patient**			
Left anterior descending artery	95	117	81.2 (72.9─87.8)
Left circumflex artery	14	17	82.4 (56.6─96.2)
Right coronary artery	37	45	82.2 (67.9─92.0)
**Previous myocardial infarction**	20	21	95.2 (76.2─99.9)
**Previous percutaneous coronary intervention**	90	108	83.3 (74.9─89.8)
**Previous coronary artery bypass grafting**	20	22	90.9 (70.8─98.9)
**≥90% stenosis in at least one coronary artery**	377	443	85.1 (81.4─88.3)
**Positive functional test result**	208	249	83.5 (78.3─87.9)
Exercise electrocardiogram	55	72	76.4 (64.9─85.6)
Stress echocardiography	2	3	66.7 (0.09─99.2)
Myocardial SPECT	95	107	88.8 (81.2─94.1)
Heart positron emission tomography	5	5	100 (54.9─100)
Heart magnetic resonance	3	3	100 (36.8─100)
Fractional flow reserve ≤0.80	66	81	81.5 (71.3─89.2)

CI, confidence interval; SPECT, single-photon emission computed tomography.

Among patients with documented positive functional test results, sensitivity was 83.5% (95% CI, 78.3–87.9). Importantly, the sensitivity was comparable between invasive and noninvasive modalities and remained similar in patients without functional testing (*Table 2*).

### Model validation on reference and external cohorts

Model validation on reference and external cohorts included 35 898 patients across four multi-institutional datasets. For positive control, we tested 788 patients (mean age 65.9 ± 9.5 years, 75.3% men) who underwent CABG for inducible ischaemia as a reference standard, and the model demonstrated a sensitivity of 86.5% (95% CI 84.0–88.9%, *Table 1*). Consistent results were demonstrated in 1764 PCI patients from IUIPH and KUDMC (*Table 1*; baseline characteristics in Supplementary material online, *Table S5*).

When tested on 12,564 healthy young individuals aged 19–35 (mean age 26.9 ± 4.2 years, 39.4% male) regarded as free of ischaemia, the model demonstrated a specificity of 92.2% (95% CI 91.8–92.7%). The AUROC and AUPRC for the combined datasets of positive and negative controls were 0.94 (95% CI 0.94─0.95) and 0.78 (95% CI 0.76─0.81), respectively. Among 20 782 patients with a negative exercise ECGs (mean age 50.3 ± 11.1 years, 60.3% men, Supplementary material online, *Table S6*), 84.4% were classified as having no inducible ischaemia.

## Discussion

In this study, we report that an AI model trained with resting 12-lead ECG effectively detects patients with inducible myocardial ischaemia, showing an AUROC of 0.90, AUPRC of 0.87, and an F1-score of 0.82. The model classified patients with inducible myocardial ischaemia from ECGs that cardiologists might find indistinguishable (*Graphical abstract*). Performance was consistent across subgroups, clinical diagnosis, or a culprit vessel. Importantly, the model demonstrated robust performance in the age- and sex-matched dataset, as well as in the various reference and external validation datasets.

ECG-trained AI has been suggested as a possible screening or diagnostic tool for various diseases.^10–15^ Some studies even validated its screening utility through prospective trials.^13–15^ While prior studies utilising ECG-trained AI to detect patients with coronary artery disease (CAD) mostly focused on detecting MI in a relatively small number of patients,^27,28^ one study developed a model to identify patients with angiographically significant coronary artery stenosis of ≥70%.^29^ However, ≥70% stenosis was demonstrated to poorly correlate with inducible myocardial ischaemia.^4,30^ In addition, as the paramount prognostic factor, identification of inducible ischaemia has been given more emphasis over the mere stenosis over time.^2,4^ Current revascularisation guidelines also favour intervention for inducible myocardial ischaemia.^31^ In this light, our study is distinct from previous studies since we focused on detecting patients with inducible ischaemia. Furthermore, recognising that the ECG fundamentally assesses cardiac electrical function, its utility is more logically aligned with detecting ischaemia-related myocardial dysfunction rather than characterising anatomical coronary stenosis, which is a structural rather than functional assessment.

In the field of AI, using large neural networks trained on both large and clean datasets is emphasized.^9,19^ With >10 000 clean data, the learning accuracy of AI can be remarkably high amidst considerable noise, even with up to 50:1 noisy-to-clean ratio.^32^ In the current study, we excluded patients with intermediate-risk or equivocal cases, thereby yielding high label fidelity and avoiding inclusion of ambiguous outcomes into the data. For instance, to maximize both the cleanliness and the size of the ‘no-ischaemia’ group, we included patients who had both 0% stenosis and CACS ≤100 in CCTA: these conditions were demonstrated to safely exclude meaningful epicardial coronary artery stenosis and inducible myocardial ischaemia, respectively.^24,25^ Additional clinical exclusion criteria further minimized inclusion of possible false negative cases. Defining the ‘ischaemia group’ also posed a challenge due to the inconsistencies among different functional tests, which are regarded as the current gold standards for identifying inducible myocardial ischaemia.^20–22^ By integrating clinical decisions made by cardiologists, along with the presence of symptoms and positive objective findings, the current approach would be one of the optimal ways to overcome an inherent limitation in defining the presence of inducible ischaemia.

Identifying inducible myocardial ischaemia is of substantial clinical value since it is prevalent and associated with adverse prognosis.^2,3^ Although it is generally encountered in patients with symptoms, asymptomatic myocardial ischaemia is also quite prevalent: it was observed not only in patients with stable CAD, but also in those with only a few risk factors for IHD, and even in apparently healthy individuals.^33,34^ Importantly, asymptomatic ischaemia significantly increased future coronary events.^33^ Evaluation for myocardial ischaemia is usually initiated by the presence of symptoms like chest pain. However, in asymptomatic subjects, routine evaluation of myocardial ischaemia is challenging, not cost-effective, and proven impractical even in those with multiple risk factors.^21,22^ Thus, it would be helpful if there were a screening tool that could effectively classify patients who require further evaluation. This is especially important since demographic changes are increasing the number of patients who have risk factors for IHD. However, currently available functional or imaging tests are costly and convey inherent risks, and are therefore not recommended by current guidelines as a general screening tool.^21,22^ Inexpensive and easy identification of high-risk groups requiring further stress or imaging tests, regardless of symptoms, can help to properly diagnose myocardial ischaemia.^5–7^ As a quick, inexpensive, non-invasive, and widely available tool, a resting 12-lead ECG analysed through a properly trained AI can be a possible candidate for a screening tool for inducible myocardial ischaemia.^10–15^ Moreover, considering comparable performance trained only with limb leads, it may also be applicable to portable devices.^10^

A comprehensive analysis of the study results might help understand some of the underlying mechanisms. First, although the models based on unmatched and age- and sex-matched datasets showed comparable performance, it is interesting to note significant differences in sensitivity and specificity in the subgroup analyses stratified by age in the unmatched model. While the high performance of the matched model confirms that the AI model based on ECG can identify ischaemia-specific signals independent of simple demographic confounders, the unmatched model, which also learned age-related risk patterns, may be more suitable for a real-world clinical practice. For instance, constructing an age- and sex-matched cohort that includes a sufficient volume of young patients is clinically unfeasible, as inducible ischaemia is extremely rare in this population. Beyond the practical necessity, applying an AI model that has not been adequately exposed to specific age groups raises significant concerns regarding generalisability and reliability. Thus, the comparative clinical utility and real-world feasibility of these models must be determined by future studies, including prospective trials. Second, our exploratory analysis demonstrated that patients who were falsely classified as positive in the ‘no-ischaemia’ group had a higher incidence of future IHD when compared with true negative cases (see Supplementary material online, *Figure S3*): for every 0.1 increase in the output value of the AI model, the likelihood of IHD increased by a factor of 1.08 (median follow-up duration 10.9 years, interquartile range 5.1─13.4; age- and sex-adjusted hazard ratio 1.08, 95% CI 1.03─1.14, *P*-value = 0.003). This suggests that the AI model might detect subclinical ischaemia even in those with minimal subclinical atherosclerosis. Third, we demonstrated a significant trend toward higher sensitivities in patients with more diseased vessels, prior revascularisation or MI. Along with the results of the exploratory analysis and acknowledging the continuum of ischaemia inducibility, these may indicate that the test results of the AI model inversely reflect the stress threshold for inducing ischaemia. Fourth, the AI might recognize subtle cell-level changes or altered ventricular hemodynamics associated with inducible ischaemia present on resting ECG. Indeed, it has been reported that patients with inducible ischaemia have elevations in biomarkers, including natriuretic peptides.^16,17^ In addition, previous studies have found an association between inducible ischaemia and abnormalities in resting ECG features, such as QT dispersion, QT peak prolongation, or increased T wave complexity.^35,36^ Although a precise interpretation remains elusive, it is plausible that the model discerns inducible ischaemia through integration of these previously reported changes in ECGs, as well as possibly as-yet-unveiled signals using deep neural structures and convolutional processes.^9,35,36^

### Limitations

Our study has some limitations. First, although we took meticulous care to define ‘ischaemia’ and ‘no-ischaemia’ groups, labelling error might exist; however, robust performance across multiple reference and external validation datasets indicates that the model was developed as intended. Second, the necessity of clearly defined case and control groups for optimal neural network training precluded the inclusion of patients with an uncertain presence of inducible ischaemia in this initial model development and testing phase. Indeed, prior work demonstrated that using only ‘learnable’ examples yields optimal performance, whereas poorly defined cases degrade accuracy.^37^ Furthermore, since the AUROC metric assumes a true binary outcome, incorporating equivocal cases would blur the class definitions and undermine this performance estimate. This inherent AI development constraint means the clinical utility of our model requires validation through future studies with distinct designs that evaluate its impact on clinical decision-making and patient outcomes. Furthermore, the established threshold value in this study needs performance optimisation for diverse clinical scenarios. For instance, although screening in the general population might be one of the clinically applicable scenarios, a significantly more conservative threshold would be necessary for the purpose of screening, because the current threshold in such a low-prevalence setting might trigger a cascade of unnecessary downstream testing, costs, and patient anxiety. Before such thresholds are set, the model output value can be provided as a continuous variable as an alternate, leaving it at the discretion of each physician to decide whether to conduct further functional tests. Third, a more in-depth analysis of the underlying mechanisms will be required. Finally, our model was developed and validated in a predominantly Asian population. Although uniform ECG criteria for diagnosis of acute MI or ischaemia generally work consistently across ethnicities, the model will need further validation in diverse ethnic groups.^11^

## Conclusions

AI trained with resting 12-lead ECG demonstrated a favourable performance in detecting patients with inducible myocardial ischaemia. It may enable the screening of high-risk patients with inducible ischaemia, potentially improving early diagnosis and risk stratification. Future studies are warranted to validate its clinical utility in various clinical settings and to assess its integration into current healthcare workflows to improve patient care.

## Supplementary Material

ztag050_Supplementary_Data

## Data Availability

The electrocardiogram data in XML format and the code of the model cannot be provided. If requested, summary-level statistics and deidentified data can be shared on reasonable request to the corresponding author, after approval from the Institutional Review Board of Seoul National University Hospital.
