# Supplementary material for: ECG trained artificial intelligence for the detection of patients with inducible myocardial ischemia
Source: Eur Heart J Digit Health. 2026 Mar 20;7(3):ztag050. doi: 10.1093/ehjdh/ztag050 (PMC13042283; doi:10.1093/ehjdh/ztag050)
Supplement: ztag050_Supplementary_Data [file ztag050_supplementary_data.docx]

**Supplemental materials**

**ECG trained Artificial Intelligence for the Detection of patients with Inducible Myocardial Ischemia**

**Short title**: ECG-trained AI for inducible myocardial ischemia

Jaehyun Lim, MD,^1,2^* Gibeom Park, MS,^3^* Hak Seung Lee, MD,^4,5^ Joon-Myoung Kwon,^4,5^ Heesun Lee, MD,^6^ Bongwon Suh, PhD^3⁑^, Hyun-Jae Kang, MD,^1,2⁑^ Yong-Jin Kim, MD,^1,2^ Bon-Kwon Koo, MD,^1,2^ Hyo-Soo Kim, MD^1,2^

^*^ These authors contributed equally to this work as the first author.

^⁑^ These authors contributed equally to this work as the corresponding author.

**Table of Contents**

Page 2  **Supplemental Methods**

Page 3-4 **Supplemental Table 1** Baseline characteristics.

Page 5  **Supplemental Table 2** Baseline characteristics of patients in an age- and sex-matched dataset.

Page 6  **Supplemental Table 3** Subgroup analysis of the test dataset.

Page 7 **Supplemental Table 4** Subgroup analysis in the test dataset of the 1:1 age- and sex-matched dataset.

Page 8 **Supplemental Table 5** Baseline characteristics of patients who received revascularization at other cardiovascular centers.

Page 9 **Supplemental Table 6** Baseline characteristics of patients with a negative exercise electrocardiogram.

Page 10 **Supplemental Figure 1** The model architecture.

Page 11  **Supplemental Figure 2** The distribution of results by ECG-trained AI.

Page 12 **Supplemental Figure 3** The Kaplan-Meier curve showing the diagnosis of ischemic heart disease between false positive and true negative patients among the no-ischemia group.

**Supplemental method 1**

**AI Model Architecture**

We utilized eight leads data from the ECG since the GE XML file format does not include all the lead information to save space without losing information: It only provides waveform data of lead I, II, and V1-6. The remaining leads (lead III, aVR, aVF, and aVL) could be reconstructed as a vector sum of leads I and II. We applied a nested convolutional neural network (CNN) structure in the classification model. CNNs are commonly applied to process images and extract features to identify objects. In this study, we considered leads as the second axis of the input, and allowed CNNs to extract both temporal and spatial features of the time-series multiple leads data.

The model was composed of five convolutional blocks that consisted of 1 convolutional layer, 1 batch normalization layer, 1 ReLU activation layer, and 1 dropout layer in sequence. Batch normalization and dropout were applied as a regularization technique to prevent overfitting. Following each convolutional block, an average pooling layer was used to summarize the features. After repeating this process for four more convolutional blocks, a max-pooling layer was employed to capture the most activated features within the window. Finally, two fully connected layers and a softmax activation layer were applied to get the probability of each class. The specific structure of the model can be represented as shown in Supplemental Figure 1, which provides a detailed depiction of the model's architecture.

**AI Model training**

First, the model was trained with ECG waveform data and related label pairs. The weights of the network were updated with the Adam optimizer and the categorical cross-entropy loss function. The model starts training on the training set with a learning rate of 1e-3. After each epoch, the entire training datasets were passed through the network, and the model was tested using the validation dataset. The validation loss of the model was checked to determine whether to continue the learning process or not. In case the loss did not improve after two epochs, the learning rate is reduced by a factor of 0.1. If there is little improvement in performance even after reducing the learning rate to 1e-6, we stopped the learning process and calculated the final performance of the model using the test dataset.

**Supplemental method 2. Constructing age- and sex-matched dataset**

This dataset was a subset of the original dataset used for the model development. However, a preliminary result of the model developed based on <5,000 age- and sex-matched subset was suboptimal, suggesting a need for a larger dataset to enhance performance. To address this issue, we further included ECGs of patients who underwent CABG for inducible ischemia, which were used for external validation, into the ischemia group. Subsequently, a 1:1 age- and sex-matched dataset comprising 7,414 cases was generated using propensity-score matching, and these data were randomly divided in an 8:1:1 ratio for training, validation, and testing of the model.

**Supplemental Table 1. Baseline characteristics**

|  | **Overall** | | **Training dataset** | | **Validation dataset** | | **Test dataset** | |
| --- | --- | --- | --- | --- | --- | --- | --- | --- |
|  | **Ischemia**  **(N=6,070)** | **No ischemia**  **(N=7,346)** | **Ischemia**  **(N=4,859)** | **No ischemia**  **(N=5,876)** | **Ischemia**  **(N=607)** | **No ischemia**  **(N=735)** | **Ischemia**  **(N=604)** | **No ischemia**  **(N=735)** |
| **Age** | 66.2±9.9 | 55.3±9.8 | 66.1±9.9 | 55.2±9.7 | 65.9±9.9 | 55.5±9.9 | 66.8±9.9 | 55.22±10.0 |
| **Sex, Men** | 4323 (71.2) | 4387 (59.7) | 3453 (71.1) | 3491 (59.4) | 436 (71.8) | 440 (59.9) | 434 (71.9) | 456 (62.0) |
| **Height (cm)** | 162.9±8.6 | 165.5±8.4 | 162.9±8.6 | 165.4±8.4 | 163.0±8.9 | 165.9±8.9 | 162.3±8.6 | 165.9±8.1 |
| **Weight (kg)** | 66.4±10.6 | 66.8±11.1 | 66.5±10.7 | 66.7±10.9 | 66.4±10.5 | 67.5±12.3 | 66.1±10.6 | 67.5±10.5 |
| **BMI (kg/m^2^)** | 25.0±3.1 | 24.3±2.9 | 25.0±3.1 | 24.3±2.8 | 25.0±3.1 | 24.4±3.0 | 25.0±3.1 | 24.5±2.8 |
| **Smoking status** | |  |  |  |  |  |  |  |
| Current smoker | 865 (14.3) | 776 (10.6) | 698 (14.4) | 615 (10.5) | 89 (14.7) | 81 (11.0) | 78 (12.9) | 80 (10.9) |
| Former smoker | 1210 (19.9) | 1282 (17.5) | 971 (20.0) | 1024 (17.4) | 116 (19.1) | 123 (16.7) | 123 (20.4) | 135 (18.4) |
| Never smoker | 3117 (51.4) | 1480 (20.1) | 2501 (51.5) | 1149 (19.6) | 315 (51.9) | 158 (21.5) | 301 (49.8) | 173 (23.5) |
| Unknown | 878 (14.5) | 3808 (51.8) | 689 (14.2) | 3088 (52.6) | 87 (14.3) | 373 (50.7) | 102 (16.9) | 347 (47.2) |
| **Hypertension** | 3733 (61.5) | 2125 (28.9) | 2980 (61.3) | 1705 (29.0) | 379 (62.4) | 211 (28.7) | 374 (61.9) | 209 (28.4) |
| **Diabetes** | 2213 (36.5) | 2146 (29.2) | 1759 (36.2) | 1738 (29.6) | 239 (39.4) | 198 (26.9) | 215 (35.6) | 210 (28.6) |
| **CKD** | 2583 (42.6) | 417 (5.7) | 2052 (42.2) | 335 (5.7) | 273 (45.0) | 39 (5.3) | 258 (42.7) | 43 (5.9) |
| **Previous PCI** | 1012 (16.7) | 0 (0.0) | 794 (16.3) | NA | 110 (18.1) | NA | 108 (17.9) | NA |
| **Previous CABG** | 164 (2.7) | 0 (0.0) | 127 (2.6) | NA | 15 (2.5) | NA | 22 (3.6) | NA |
| **Previous MI** | 185 (3.1) | 0 (0.0) | 148 (3.1) | NA | 16 (2.6) | NA | 21 (3.5) | NA |
| **Previous CHF** | 79 (1.3) | 156 (2.1) | 67 (1.4) | 128 (2.2) | 7 (1.2) | 10 (1.4) | 5 (0.8) | 18 (2.4) |
| **Previous CVA** | 365 (6.0) | 487 (6.6) | 286 (5.9) | 379 (6.4) | 42 (6.9) | 50 (6.8) | 37 (6.1) | 58 (7.9) |
| **Ejection fraction (%)** | 60.1 (8.1) | 64.1 (6.5) | 60.1±8.2 | 64.1±6.5 | 60.3 (7.8) | 64.0 (6.5) | 59.9 (7.7) | 64.1 (6.3) |
| **Ischemia group specific characteristics** | |  |  |  |  |  |  |  |
| **Clinical diagnosis** |  |  |  |  |  |  |  |  |
| silent ischemia | 793 (13.1) | NA | 642 (13.2) | NA | 69 (11.4) | NA | 79 (13.1) | NA |
| stable angina | 3,488 (57.5) | NA | 2,791 (57.4) | NA | 357 (58.8) | NA | 345 (57.1) | NA |
| unstable angina | 1,791 (29.5) | NA | 1,426 (29.3) | NA | 181 (29.8) | NA | 180 (29.8) | NA |
| **n-vessel disease** |  |  |  |  |  |  |  |  |
| 1-vessel disease | 1,910 (31.5) | NA | 1,545 (31.8) | NA | 187 (30.8) | NA | 178 (29.5) | NA |
| 2-vessel disease | 1,863 (30.7) | NA | 1,462 (30.1) | NA | 202 (33.3) | NA | 199 (32.9) | NA |
| 3-vessel disease | 1,722 (28.4) | NA | 1,382 (28.4) | NA | 157 (25.9) | NA | 183 (30.3) | NA |
| Left main disease | 575 (9.4) | NA | 470 (9.7) | NA | 61 (10.0) | NA | 44 (7.3) | NA |

BMI, body mass index; CABG, coronary artery bypass grafting; CHF, congestive heart failure; CKD, chronic kidney disease; CVA, cerebrovascular accident; MI, myocardial infarction; NA, not applicable; PCI, percutaneous coronary intervention

**Supplemental Table 2. Baseline characteristics of patients in an age- and sex-matched dataset.**

|  | **Training dataset** | | **Validation dataset** | | **Test dataset** | |
| --- | --- | --- | --- | --- | --- | --- |
|  | **No ischemia group (N=2,225)** | **Ischemia group (N=2,225)** | **No ischemia group (N=741)** | **Ischemia group (N=741)** | **No ischemia group (N=741)** | **Ischemia group(N=741)** |
| Age, years | 60.5±8.7 | 60.4±8.7 | 60.7±8.9 | 60.7±8.9 | 60.4±8.4 | 61.0±8.9 |
| Sex, male | 1519 (68.3%) | 1412 (66.4%) | 495 (66.8%) | 497 (67.4%) | 499 (67.7%) | 499 (67.7%) |
| Body mass index, kg/m^2^ | 24.4±2.8 | 25.2±3.1 | 24.2±2.7 | 25.2±3.0 | 24.6±2.8 | 25.3±3.0 |
| Hypertension | 704 (31.7%) | 1131 (57.7%) | 262 (35.4%) | 389 (59.0%) | 213 (28.7%) | 397 (59.9%) |
| Diabetes mellitus | 648 (29.1%) | 703 (35.9%) | 213 (28.7%) | 218 (33.1%) | 212 (28.6%) | 244 (36.8%) |
| Previous myocardial infarction | NA | 58 (3.0%) | NA | 17 (2.6%) | NA | 14 (2.1%) |
| Clinical diagnosis of the ischemia group |  |  |  |  |  |  |
| silent ischemia | NA | 287 (13.5%) | NA | 94 (12.8%) | NA | 98 (13.3%) |
| stable angina | NA | 1203 (56.6%) | NA | 427 (57.9%) | NA | 431 (58.5%) |
| unstable angina | NA | 636 (29.9%) | NA | 216 (29.3%) | NA | 208 (28.2%) |

NA, not applicable

**Supplemental Table 3. Subgroup analysis of the test dataset**

|  | **Sensitivity (%)** | **Specificity (%)** | **AUROC (95% CI)** |
| --- | --- | --- | --- |
| **Total** | 83.8 (506/604) | 79.6 (585/735) | 0.90 (0.88─0.91) |
| **Sex** |  |  |  |
| Men | 83.9 (364/434) | 78.3 (357/456) | 0.89 (0.87─0.92) |
| Women | 83.5 (142/170) | 81.7 (228/279) | 0.90 (0.87─0.93) |
| **Age group, years** |  |  |  |
| < 60 | 73.9 (105/142) | 84.6 (433/512) | 0.88 (0.85─0.91) |
| ≥ 60 | 86.8 (401/462) | 68.2 (152/223) | 0.86 (0.83─0.89) |
| **Hypertension** |  |  |  |
| Yes | 84.2 (315/374) | 78.0 (163/209) | 0.90 (0.87─0.92) |
| No | 83.0 (191/230) | 80.2 (422/526) | 0.89 (0.87─0.92) |
| **Diabetes mellitus** |  |  |  |
| Yes | 85.6 (184/215) | 80.0 (168/210) | 0.92 (0.89─0.94) |
| No | 82.8 (322/389) | 79.4 (417/525) | 0.89 (0.87─0.91) |

AUROC, area under the receiver operating characteristic curve; CI, confidence interval

**Supplemental Table 4. Subgroup analysis in the test dataset of the 1:1 age- and sex-matched dataset.**

|  | **Sensitivity (%)** | **Specificity (%)** | **AUROC (95% CI)** |
| --- | --- | --- | --- |
| **Total** | 81.4 (603/741) | 70.9 (525/741) | 0.85 (0.83─0.87) |
| **Sex** |  |  |  |
| Men | 81.0 (404/499) | 72.7 (363/499) | 0.86 (0.84─0.88) |
| Women | 81.9 (195/238) | 66.9 (162/242) | 0.82 (0.78─0.86) |
| **Age group, years** |  |  |  |
| < 60 | 81.0 (264/326) | 73.0 (243/333) | 0.86 (0.83─0.89) |
| ≥ 60 | 81.5 (335/411) | 69.1 (282/408) | 0.84 (0.81─0.87) |
| **Hypertension** |  |  |  |
| Yes | 80.9 (321/397) | 69.0 (147/213) | 0.84 (0.81─0.88) |
| No | 82.0 (282/344) | 71.6 (378/528) | 0.85 (0.82─0.87) |
| **Diabetes mellitus** |  |  |  |
| Yes | 84.8 (207/244) | 68.9 (146/212) | 0.86 (0.82─0.89) |
| No | 79.7 (396/497) | 71.6 (379/529) | 0.84 (0.82─0.87) |

AUROC, area under the receiver operating characteristic curve; CI, confidence interval

**Supplemental Table 5. Baseline characteristics of patients who received revascularization at other cardiovascular centers.**

|  | **KUDMC (N=1,701)** | **IUIPH (N=63)** |
| --- | --- | --- |
| Age, years | 64.8±9.7 | 67.3±13.2 |
| Sex, Men | 1124 (66.1%) | 46 (73.0%) |
| Height (cm) | 161.9±9.0 | 163.9±9.2 |
| Weight (kg) | 65.6±11.2 | 66.0±10.5 |
| Body mass index (kg/m^2^) | 24.9±3.3 | 24.5±2.7 |
| Current smoking | 415 (24.4%) | 23 (36.5%) |
| Hypertension | 946 (55.6%) | 39 (61.9%) |
| Diabetes mellitus | 569 (33.5%) | 32 (50.8%) |
| Chronic kidney disease | 61 (3.6%) | 5 (7.9%) |
| Previous PCI | 207 (12.2%) | 8 (12.7%) |
| Previous CABG | 0 (0.0%) | 0 (0.0%) |
| Previous MI | 31 (1.8%) | 3 (4.8%) |
| Previous CHF | 590 (34.7%) | 3 (4.8%) |
| Previous CVA | 147 (8.6%) | 9 (14.3%) |
| **Clinical diagnosis** |  |  |
| silent ischemia | 33 (1.9%) | NA^*^ |
| stable angina | 1134 (66.7%) | NA^*^ |
| unstable angina | 534 (31.4%) | NA^*^ |
| **n-vessel disease** |  |  |
| 1-vessel disease | 712 (41.8%) | 25 (39.7%) |
| 2-vessel disease | 501 (29.5%) | 19 (30.2%) |
| 3-vessel disease | 306 (18.0%) | 18 (28.6%) |
| Left main disease | 182 (10.7%) | 1 (1.6%) |

CABG, coronary artery bypass grafting; CHF, congestive heart failure; CVA, cerebrovascular accident; IUIPH, Inje University Ilsan Paik Hospital; KUDMC, Keimyung University Dongsan Medical Center; MI, myocardial infarction; NA, not applicable; PCI, percutaneous coronary intervention

* Clinical diagnoses for patients at IUIPH were not obtained. However, high-sensitivity troponin I levels were measured for every patient before percutaneous coronary intervention, and those with levels >99^th^ percentile were considered to have myocardial infarction and subsequently excluded.

**Supplemental Table 6. Baseline characteristics of patients with a negative exercise electrocardiogram.**

|  | **Negative exercise electrocardiogram (N=20,782)** |
| --- | --- |
| Age (years) | 50.3±11.1 |
| Sex, Men | 12532 (60.3%) |
| Height (cm) | 167.3±8.3 |
| Weight (kg) | 66.7±12.8 |
| Body mass index (kg/m^2^) | 23.6±3.5 |
| Current smoking | 5302 (25.5%) |
| Hypertension | 3827 (18.4%) |
| Diabetes mellitus | 2281 (11.0%) |
| Chronic kidney disease | 346 (1.7%) |
| Previous percutaneous coronary intervention | NA |
| Previous coronary artery bypass grafting | NA |
| Previous myocardial infarction | NA |
| Previous congestive heart failure | 0 (0.0%) |
| Previous cerebrovascular accident | 77 (0.4%) |

NA, not applicable

**Supplemental Figure 1. The model architecture.**

**
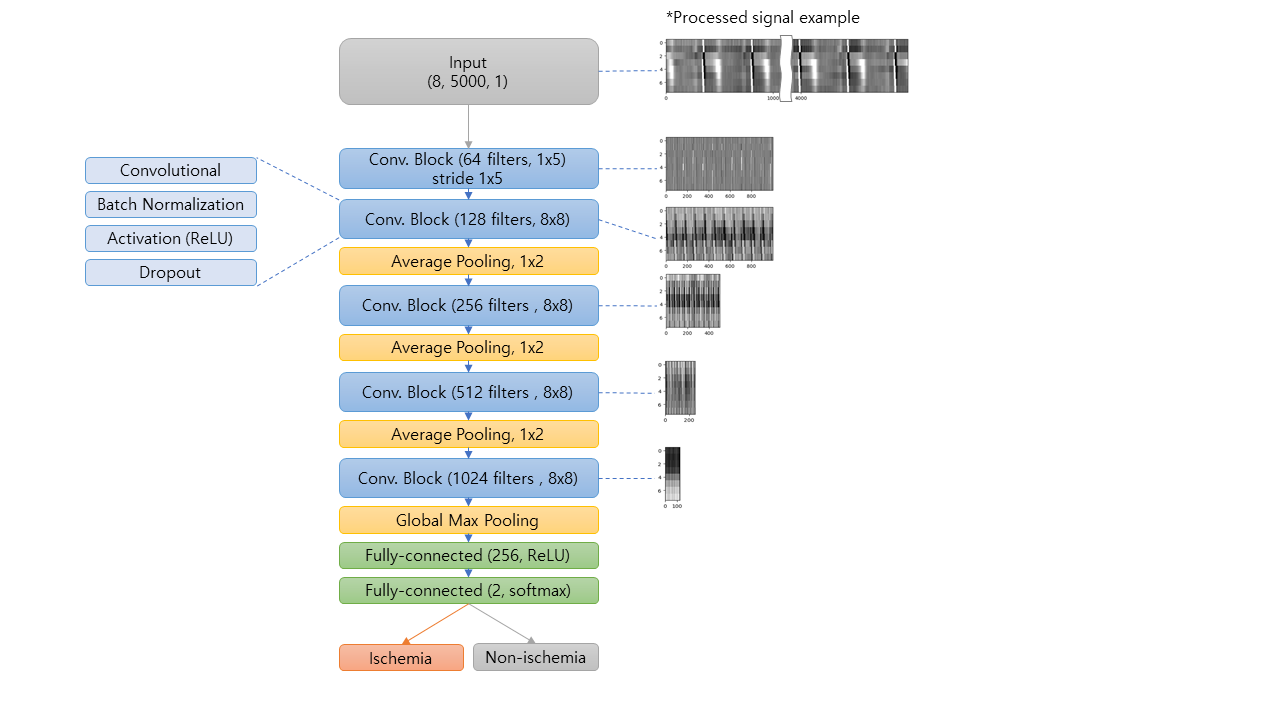
**

**Supplemental Figure 2. The distribution of results by ECG-trained AI.**

**
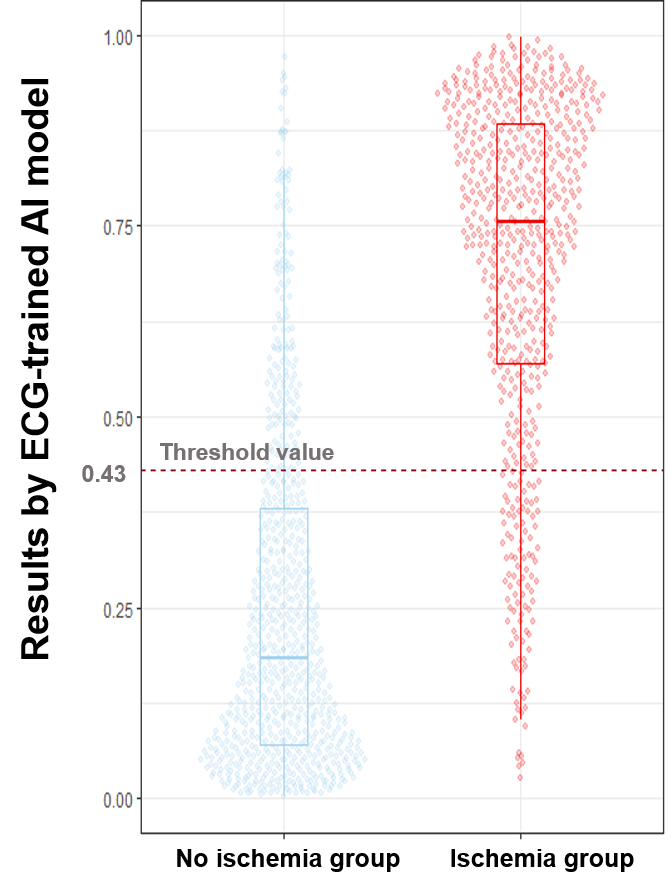
**

AI, artificial intelligence; ECG, electrocardiogram.

**Supplemental Figure 3. The Kaplan-Meier curve showing ischemic heart disease-free survival between false positive and true negative patients among the no-ischemia group.**

**
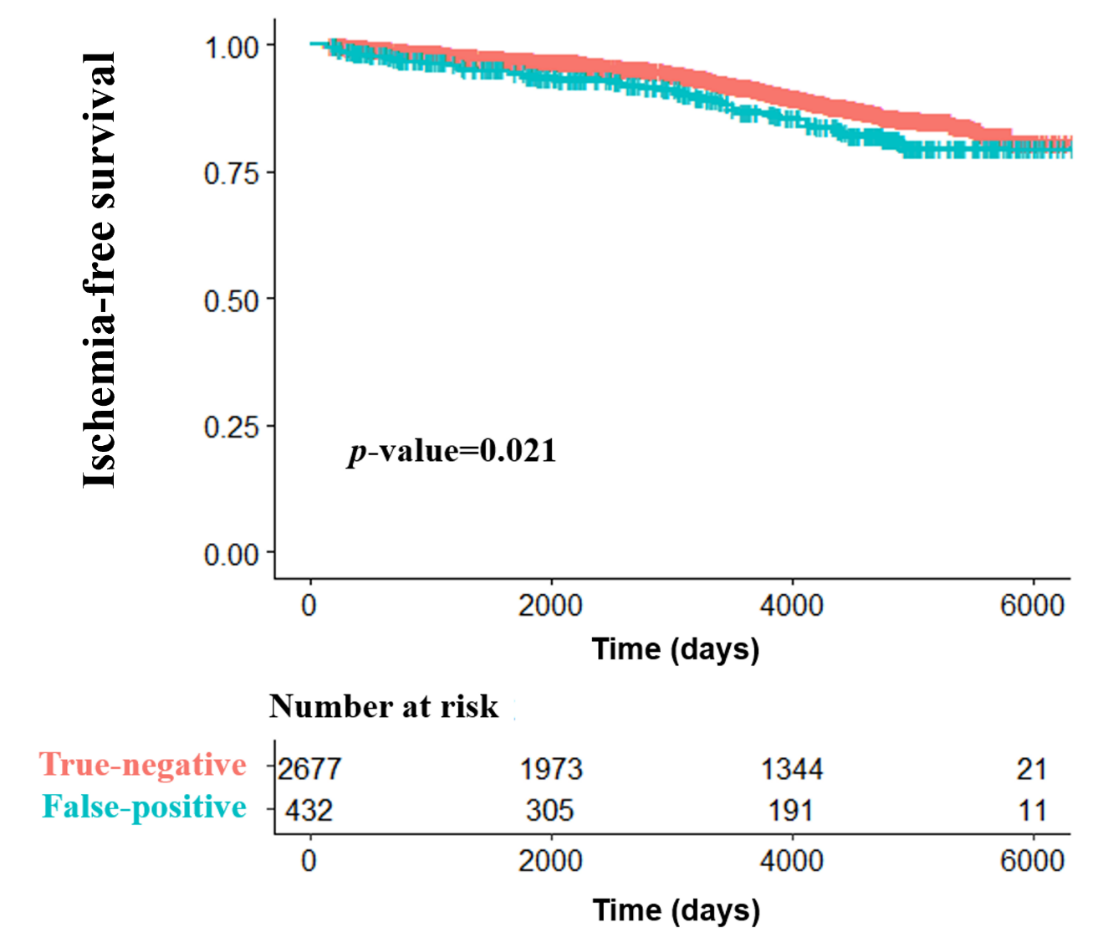
**

An exploratory analysis comparing false-positive and true-negative patients in the no-ischemia group. Although both groups of patients had 0% stenosis in coronary computed tomography angiography and coronary calcium score of less than 100, patients who were falsely classified as positive by artificial intelligence model were more likely to be diagnosed of ischemic heart disease during follow up.
